# Novel Small Molecule Inhibitors That Prevent the Neuroparalysis of Tetanus Neurotoxin

**DOI:** 10.3390/ph14111134

**Published:** 2021-11-08

**Authors:** Giulia Zanetti, Andrea Mattarei, Florigio Lista, Ornella Rossetto, Cesare Montecucco, Marco Pirazzini

**Affiliations:** 1 Department of Biomedical Sciences, University of Padova, Via U. Bassi 58/B, 35121 Padova, Italy; giulia.zanetti@unipd.it (G.Z.); ornella.rossetto@unipd.it (O.R.); 2 Department of Pharmaceutical and Pharmacological Sciences, University of Padova, Via F. Marzolo 5, 35131 Padova, Italy; andrea.mattarei@unipd.it; 3 Scientific Department, Army Medical Center, Via Santo Stefano Rotondo 4, 00184 Rome, Italy; romano.lista@gmail.com; 4 Italian Research Council, Institute of Neuroscience, University of Padova, Via U. Bassi 58/B, 35121 Padova, Italy; 5 CIR-Myo, Centro Interdipartimentale di Ricerca di Miologia, University of Padova, Via U. Bassi 58/B, 35131 Padova, Italy

**Keywords:** tetanus neurotoxin, trafficking, disulfide reduction, thioredoxin system inhibitors, EGA

## Abstract

Tetanus neurotoxin (TeNT) is a protein exotoxin produced by *Clostridium tetani* that causes the deadly spastic neuroparalysis of tetanus. It consists of a metalloprotease light chain and of a heavy chain linked via a disulphide bond. TeNT binds to the neuromuscular junction (NMJ) and it is retro-axonally transported into vesicular compartments to the spinal cord, where it is released and taken up by inhibitory interneuron. Therein, the catalytic subunit is translocated into the cytoplasm where it cleaves its target protein VAMP-1/2 with consequent blockage of the release of inhibitory neurotransmitters. Vaccination with formaldehyde inactivated TeNT prevents the disease, but tetanus is still present in countries where vaccination coverage is partial. Here, we show that small molecule inhibitors interfering with TeNT trafficking or with the reduction of the interchain disulphide bond block the activity of the toxin in neuronal cultures and attenuate tetanus symptoms in vivo. These findings are relevant for the development of therapeutics against tetanus based on the inhibition of toxin molecules that are being retro-transported to or are already within the spinal cord and are, thus, not accessible to anti-TeNT immunoglobulins.

## 1. Introduction

Tetanus neurotoxin (TeNT) is produced by *Clostridium* *tetani* and together with botulinum neurotoxins (BoNTs) forms the large, and still growing, family of Clostridial Neurotoxins (CNTs) [1,2,3]. The CNTs are the etiological agents of botulism (BoNTs) and tetanus (TeNT), two deadly neuroparalytic syndromes affecting vertebrates characterized by a flaccid and a spastic paralysis, respectively. They are the most poisonous substances known to mammalians with lethal doses in the low ng/kg range [4]. Such a potency derives from their ability to block enzymatically neurotransmission, which is an essential neurophysiological function.

BoNTs and TeNT have similar structures consisting of a 100 kDa heavy chain (H) and a 50 kDa catalytically active light chain (L) linked via a single interchain disulphide bridge [5]. The opposite symptoms of flaccid and spastic paralysis solely depend on BoNTs and TeNT targeting different neurons. This is dictated by the carboxyl-terminal fragment of H (HC) [6,7] that binds the presynaptic membrane at the neuromuscular junction (NMJ) and determines a different trafficking of BoNTs and TeNT within motor axon terminals. BoNTs are locally internalized [8,9], while TeNT ends inside endosomal vesicles that are retro-transported along the axons of alpha-motor neurons up to the perikaryon inside the spinal cord [10,11]. Thereafter, TeNT is released, binds, and enters inhibitory interneurons similarly to BoNTs at the NMJ [12]. In fact, both TeNT and BoNTs are internalized into susceptible neurons via synaptic vesicles (SV) [13,14] and translocate their catalytic L chain into the cytosol following a conformational change of the N-terminal half of the H chain triggered by the acidification of the SV lumen [15,16]. After membrane translocation, the interchain disulphide bond of BoNTs and TeNT is reduced by the NADPH–Thioredoxin Reductase–Thioredoxin (TrxR–Trx) system, [17,18,19,20,21,22]. This step leads to the release of the L chain from the SV surface into the cytosol [23,24], thus enabling their catalytic activity [24,25]. Within the cytosol, the L metalloproteases selectively cleaves specific members of the SNARE protein family [3,5,25,26], which are essential constituents of the SV neurotransmitter release machinery [27]. In the case of TeNT, the cleavage of VAMP-1/2 (also known as synaptobrevin-1/2) blocks the release of GABA and glycine from inhibitory interneurons of the spinal cord, which prevents the balanced contraction of opposing skeletal muscles and causes a spastic paralysis with contractures and uncontrollable muscle spasms [12,28,29,30,31]. This is accompanied by autonomic dysregulation and respiratory failure that can lead to death [32,33,34].

Currently, tetanus is effectively prevented by vaccination with tetanus toxoid or by passive immunization with anti-TeNT immune-globulins (TIGs) as a prophylaxis to neutralize circulating toxins [35]. Moreover, an intense effort is underway to develop highly purified human monoclonal antibodies [36,37,38,39,40,41], which overcome some drawbacks associated with the use of TIG [36]. Nonetheless, tetanus remains a major killer in many countries where the availability of anti-tetanus vaccine and of antisera are limited [35,42,43,44]. In addition, TIG is administered intramuscularly and, thus, provides a spectrum of TeNT neutralization restricted to peripheral body fluids. While sufficient for prophylaxis, this administration protocol does not allow TIG to reach and block the toxin molecules already internalized into peripheral nerves limiting its effectiveness in symptomatic tetanus [45].

This situation calls for the development of alternative strategies [45]. We have recently shown that the neurotoxicity of BoNTs can be potently attenuated by small molecules drugs interfering with their mechanism of action [45]. These include Thioredoxin Reductase-Thioredoxin (TrxR–Trx) inhibitors that block the reduction of the interchain disulphide bond and 4-bromobenzaldehyde N-(2,6-dimethylphenyl) semicarbazone (EGA), which interferes with BoNT trafficking at the nerve terminals [20,46,47].

Based on the similarity of structure and nerve terminal action of TeNT and BoNTs, we analyzed the impact of TrxR–Trx inhibitors and EGA on TeNT intoxication and tested the possibility that these small molecule inhibitors of BoNTs can be used for the prevention of the neuroparalytic action of TeNT.

We show here that TrxR–Trx inhibitors and EGA effectively prevent the toxicity of TeNT on neuronal cultures and, more importantly, decrease tetanus symptoms severity in mice. Altogether, these results suggest that compounds that interfere with disulphide reduction or toxin trafficking can be used to attenuate TeNT intoxication and reduce tetanus severity.

## 2. Results

### 2.1. Inhibitors of the Thioredoxin Reductase–Thioredoxin Redox System Prevent the TeNT-Induced Cleavage of VAMP-2 in Cultured Neurons

Following the demonstration that the cytosolic reduction of the interchain disulphide bond of CNTs is essential to enable their metalloprotease activity [23,24] and the recent finding that TrxR–Trx inhibitors prevent the toxicity of all BoNT serotypes [45], we investigated whether TrxR–Trx inhibitors prevent TeNT intoxication in vitro.

**Figure 1:**
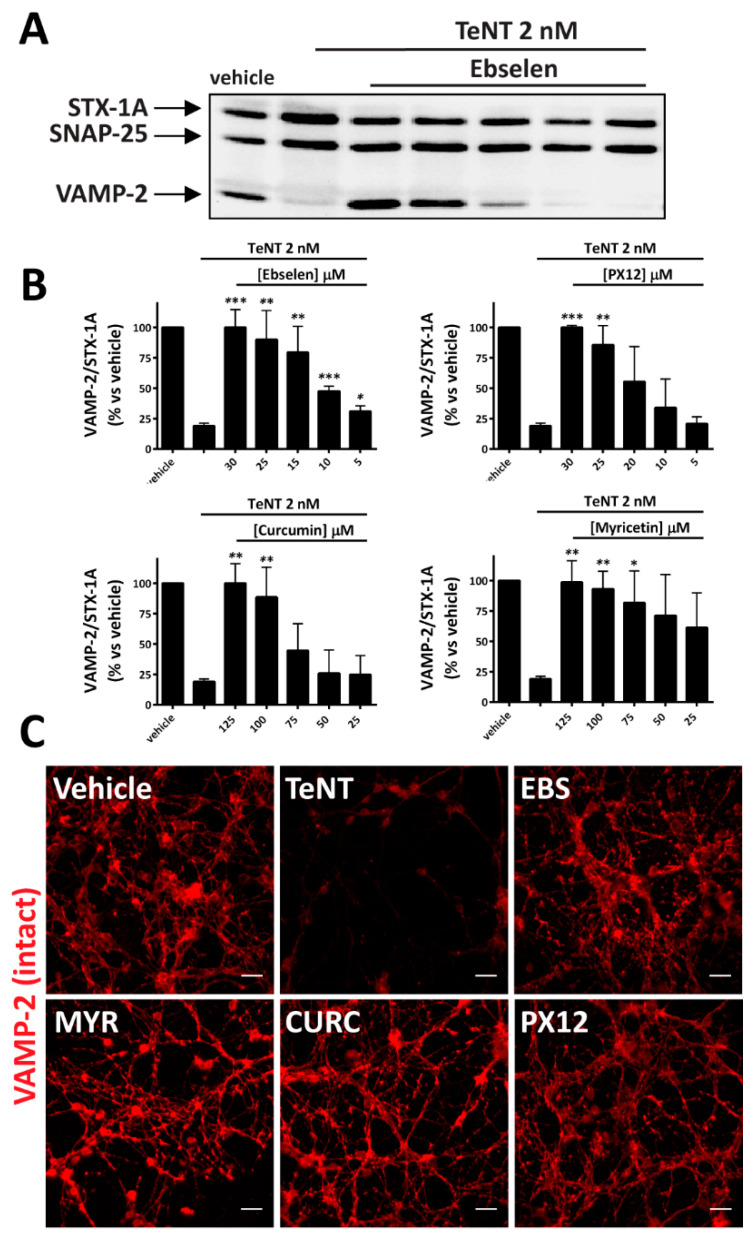
Thioredoxin Reductase–Thioredoxin (TrxR–Trx) inhibitors prevent TeNT cleavage of VAMP-2 in cultured neurons. CGNs were incubated with the indicated concentration of inhibitors at 37 °C for 30 min. TeNT 2 nM was added for 10 min, cells were washed, and culture medium with the same concentration of inhibitors was restored. Incubation was then prolonged for 4 h at 37 °C. (**A**) Representative immunoblot showing the relative amount of VAMP-2, SNAP-25, and Syntaxin-1A in CGNs in control condition (vehicle), upon treatment with TeNT alone, and upon pre-treatment with decreasing amounts (empty triangle) of Ebselen before TeNT addition. VAMP-2 content was estimated with an antibody recognizing the intact form of VAMP-2 and STX-1A signal was used as loading control. (**B**) Quantification of experiments performed, such as in (**A**), with the indicated inhibitors. SD values derived from at least three independent experiments. Graphs show mean ± SD. Significance was calculated by a two tailed Student’s *t*-test compared to neurons treated with TeNT alone (*** *p* < 0.001; ** *p* < 0.01; * *p* < 0.05) (**C**) Representative immunofluorescence for VAMP-2 staining in CGNs treated as in (**A**). Vehicle shows the control condition (no toxin added). Inhibitors were used as follows: Ebselen 30 µM, PX12 30 µM, curcumin 125 µM, and myricetin 125 µM. Panels are representative of three independent experiments. Scale bar is 20 µm.

A convenient way to screen the inhibitory activity of chemical compounds against CNTs toxicity is visualization of cleavage of their substrates using cerebellar granular neurons (CGNs) which are very sensitive to TeNT [48]. Figure 1A shows that TeNT cleaves almost completely its substrate VAMP-2, as evaluated by Western blotting with a specific antibody recognizing the intact form of the protein. At the same time, pre-treatment of CGNs with inhibitors of Trx (Ebselen, PX12) or of TrxR (curcumin and myricetin) prevents the cleavage of VAMP-2 in a dose-dependent manner (Figure 1A,B). This protective effect can be observed also by monitoring substrate cleavage by immunofluorescence. As shown in Figure 1C, VAMP-2 is highly expressed in CGNs where it is predominantly located within neuronal processes. TeNT treatment causes the almost complete loss of this signal, which is instead preserved by pre-treating CGNs with TrxR–Trx inhibitors. Notably, the concentration range whereby each inhibitor prevents TeNT toxicity is similar to that previously reported to protect CGNs from the neurotoxicity of several BoNT serotypes [20,45].

The comparable inhibition profiles between TeNT and BoNTs indicate that these toxins are similarly susceptible to the reduction of the interchain disulphide bond by the TrxR–Trx system and that this molecular step represents a pharmacological target to block the activity of TeNT as reported for BoNTs [20,45].

### 2.2. EGA Prevents TeNT-Induced Cleavage of VAMP-2 in Cultured Neurons

Considering the importance of CNTs trafficking upon entry into nerve terminals, we also tested EGA, a compound that was found to interfere with the intracellular vesicular trafficking of several pathogens [47,49,50,51].

**Figure 2:**
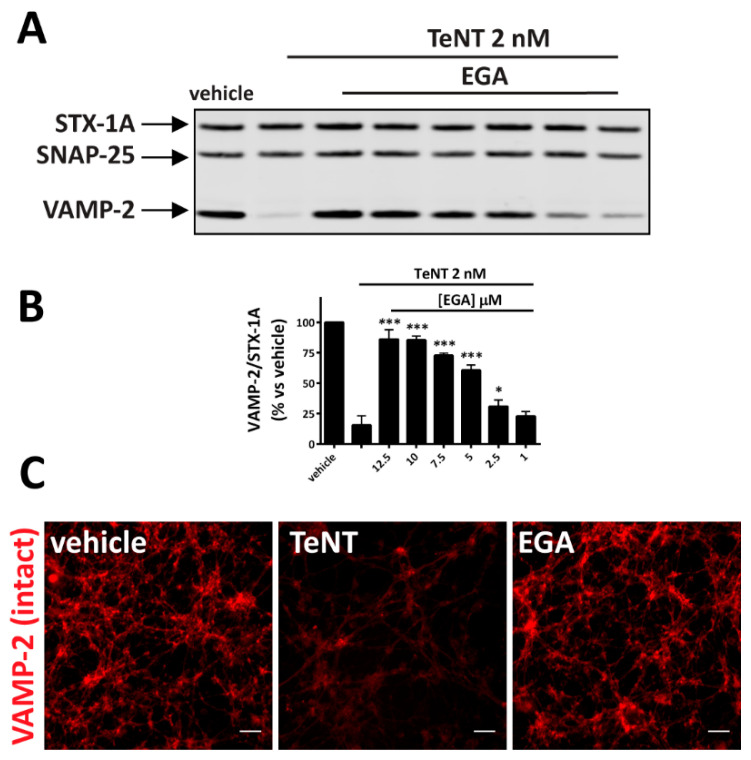
EGA prevents the TeNT-induced cleavage of VAMP-2 in cultured neurons. CGNs were incubated with different concentrations of EGA at 37 °C for 30 min. TeNT 2 nM was added for 10 min, cells were washed, and culture medium with the same concentration of inhibitor was restored. Incubation was then prolonged for 4 h at 37 °C. (**A**) Representative immunoblot showing the relative amount of VAMP-2, SNAP-25 and Syntaxin-1A in CGNs in control conditions (vehicle), upon treatment with TeNT alone (PC) and upon pre-treatment with decreasing amounts of EGA before TeNT addition. (**B**) Quantification of VAMP-2 signal in Western blots reported as a percent vs. vehicle and calculated as a ratio of STX-1A staining used as loading. SD values derive from three independent experiments. Graphs show mean ± SD. Significance was calculated by a two tailed Student’s *t*-test compared to neurons treated with TeNT alone (*** *p* < 0.001; * *p* < 0.05) (**C**) Representative immunofluorescence for VAMP-2 staining in CGNs treated as in (**A**). EGA was used at 12.5 µm. The image is representative of three independent experiments. Scale bar is 20 µm.

Figure 2A,B show that EGA protects CGNs from TeNT activity against VAMP-2. As assessed by Western blot and immunofluorescence analyses, also in this case the inhibition is dose-dependent and occurs in a concentration range of EGA similar to that previously reported to block the activity of BoNTs in the same neuronal culture model [47].

### 2.3. Ebselen and EGA Attenuate Generalized Tetanus In Vivo

**Figure 3:**
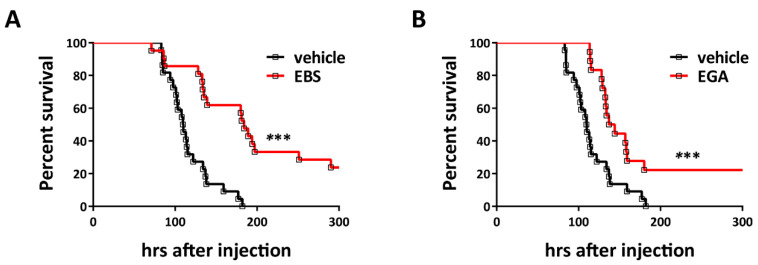
Ebselen and EGA attenuate generalized tetanus in mice. Adult male CD1 mice were pre-treated with (**A**) Ebselen 7.5 mg/kg (*n* = 21) or (**B**) EGA 20 mg/kg (*n* = 19) or vehicle (*n* = 22) as described in Section 4. Thereafter, a double LD_50_ of TeNT were i.p.-injected. Animals were monitored every 6 h for 15 days, after which the experiment was considered concluded. Graphs show the survival rate curves. Significance between the curves was calculated using the Gehan–Breslow–Wilcoxon test. (Ebselen: *p* = 0.0002 *** and EGA *p* = 0.0005 ***).

To test whether these small molecule drugs can prevent TeNT activity also in vivo, we assayed their activity in mice i.p. inoculated with the toxin to cause generalized tetanus. Here, we limited our analysis to Ebselen and EGA to reduce the number of animals for ethical reasons. Figure 3 shows that 2 × LD_50_ of TeNT induces the development of systemic tetanus in a synchronous manner, with mice displaying the symptoms of spastic paralysis in a narrow time window (black trace). At variance, the red traces of Figure 3A,B show that Ebselen or EGA pre-treatment strongly protects mice from TeNT neurotoxicity. Notably, mice displayed a significant delay in the development of tetanus symptoms when compared to mice injected only with TeNT. Such an attenuation in the severity of TeNT intoxication by Ebselen and EGA also resulted in a lower overall death rate, with survivor animals fully recovering from symptoms in a few days.

The drugs used here did not achieve full protection, possibly because their amount within the spinal cord may have been reduced. However, these data suggest that a large amount of injected TeNT did not reach its target VAMP-2 in susceptible neurons in the spinal cord, reinforcing the suggestion that the perturbation of TeNT mechanism of toxicity may represent a valuable pharmacological target to develop anti-tetanus drug, as it was for BoNTs [20,22,45,46].

## 3. Discussion

Here, we provided experimental evidence that TeNT neurotoxicity can be contrasted by using small molecule drugs interfering with its mechanism of nerve terminal intoxication. We first found that inhibitors of TrxR–Trx (Ebselen, myricetin, curcumin, and PX12) prevent TeNT cleavage of VAMP-2 in primary neuronal cultures. Together with the previous finding that the TrxR–Trx system specifically reduces the interchain disulphide of TeNT and BoNTs in vitro [17,18], this result suggests that TrxR–Trx inhibitors prevent the release of TeNT light chain into the neuronal cytosol as recently shown for BoNTs. Most importantly, we found that Ebselen, which is an inhibitor of both Trx and TrxR, is effective in preventing the development of generalized tetanus in mice. This finding extends our previous results showing that Trx–TrxR inhibitors are effective anti-botulism drugs [20,21,45] and strongly indicate that the reduction of the interchain disulphide bond is an essential common step to enable the intracellular protease activity of all CNTs.

The second finding presented here is that EGA, which prevents the activity of several pathogens requiring a passage through an acidic compartment by interfering with the endosomal trafficking route [47,49,50,51], blocks also the activity of TeNT on CGNs. This result is in line with the notion that TeNT requires the entry into the lumen of and intracellular acidic compartment to translocate its L chain into the cytosol [16], and it suggests that EGA very likely interferes with TeNT trafficking within the nerve terminals of their target neurons, i.e., inhibitory interneurons of the spinal cord. Even though the molecular target of EGA is not known, its similar effect on TeNT and BoNT intoxication strongly suggests that these toxins may be trafficked through a common compartment preceding the translocation of the L chain into the cytosol. This possibility is in keeping with the observation that TeNT, like BoNTs, is internalized into its target neurons via synaptic vesicles recycling [8,13,14]. This would be an indirect indication that EGA is capable of reaching the spinal cord intercellular liquid. However, it cannot be excluded that EGA also interferes with the entry of TeNT into signaling endosomes at the NMJ thus affecting its retro-axonal transport toward the spinal cord.

Altogether, the present results add to the conclusion that, despite causing strikingly different diseases, TeNT and BoNTs act similarly at cellular level and that drugs impinging on the same neuronal entry step will act similarly independently on the TeNT and BoNT types considered. This conclusion is even more important if one considers the large number of novel BoNTs that are being discovered [1,2,52] and the number of tetanus cases still afflicting people worldwide [31,44].

Currently, despite the reduced cost of the very effective tetanus vaccine, its availability and/or the vaccination programs in some areas of the world remain inadequate, with tetanus still being a major killer, particularly in the first month of life [12,24,34]. In fact, a horrible form of tetanus is *tetanus neonatorum*, which affects neonates born from un-vaccinated mothers and is caused by the use of non-sterile surgical tools to cut the umbilical cord or by the use of tetanus spore contaminated materials to cauterize wounds; *tetanus neonatorum* is characterized by a high mortality rate [53,54].

In addition, despite being one of the most successful public health achievements, a significant part of the population of high income countries currently perceives vaccination as unsafe and unnecessary, rejecting vaccines [55], and this is bound to lead to increasing tetanus prevalence.

Accordingly, this situation calls for the development of alternative strategies. When symptoms of tetanus (e.g., muscles contractures and spasms) become evident, there are no specific drugs able to block the pathology but the administration of TIG to neutralize circulating toxins [24]. However, once TeNT is internalized into peripheral nerves to be retro-transported in the central nervous system, they are no longer effective as immunoglobulins cannot enter neurons. Although the inhibitors tested here cannot rescue the intoxicated neurons from the TeNT molecules already acting inside inhibitory interneurons, they hold a remarkable advantage, which is the ability to prevent the toxicity of the TeNT molecules still travelling inside peripheral nerves and not having yet reached inhibitory interneurons. In fact, this “travelling population”, which is no longer in body fluids but has already been taken up by peripheral nerve terminals, cannot be neutralized by anti-TeNT immunoglobulins. In terms of therapeutic outcome, the importance of blocking this intraneural pool of TeNT molecules is demonstrated by the fact that TIG is more effective when injected intrathecally. Nonetheless, this procedure is not recommended due to the amount of protein that can be safely injected in the cerebrospinal fluid [56,57]. Therefore, the use of these molecules is expected to potentiate the effect of intramuscular TIG, which can have a major impact on humans who have long peripheral nerves able to take up large quantities of TeNT molecules. Given that the severity and duration of the pathological signs correlate with the amount of toxin that reaches the interneuron cytosol of inhibitory interneurons in the spinal cord, Ebselen and EGA hold the potential to significantly improve the therapeutic effectiveness of TIG and to influence the duration of tetanus symptoms, thus shortening the period of hospitalization and reducing the high costs associated with intensive care.

Altogether, our results suggest that compounds that interfere with disulphide reduction or toxin trafficking could be used in clinics to improve prophylaxis, also considering the lower production cost and the greater stability of these molecules which is particularly important in low-income countries, where tetanus affects large number of patients, particularly children. Therefore, they can be considered lead compounds to develop a common strategy to prevent/treat all CNTs intoxication by targeting their mechanism of action.

## 4. Materials and Methods

### 4.1. Reagents

Cytosine β-D-arabinofuranoside hydrochloride (C6645), DNase I from bovine pancreas (DN25), poly-L-lysine hydrobromide (P1274) and trypsin (T4799) were purchased from Sigma Aldrich (St. Louis, Missouri, USA).

Primary antibodies: anti-SNAP-25 (SMI81, ab24737) was purchased from Abcam(Cambridge, UK), while anti-syntaxin-1A (STX-1A, 110111) and anti-VAMP-2 were purchased from Synaptic System (Göttingen, Germany). Secondary antibodies: HRP-conjugated Ab were purchased from Calbiochem^®^ (San Diego, CA, USA), Alexa Fluorophores 488- or 555-conjugated Ab were from Thermo-Fisher Scientific (Waltham, MA, USA). Native TeNT was purified as previously described [58].

Inhibitors: myricetin [3,3′,4′,5,5′7-Hexahydroxyflavone] and Curcumin [(E,E)-1,7-bis(4-Hydroxy-3- methoxyphenyl)-1,6-heptadiene-3,5-dione] were purchased from Sigma Aldrich. PX12 [2-[(1-Methylpropyl) dithio]-1H-imidazole] and Ebselen [2-Phenyl-1,2-benzisoselenazol3(2H)-one] were purchased from Santa Cruz Biotechnology (Dallas, TX, USA). 4-Bromobenzaldehyde N-(2,6-dimethylphenyl) semicarbazone (EGA) was synthesized as in [47].

### 4.2. Neuronal Cultures and Intoxication Assay

Primary cultures of rat cerebellar granule neurons are highly pure (more than 95%) and homogeneous (mostly granule cells) cultures model. They were prepared from 6 to 8 day old rats as previously described [48]. Briefly, cerebella were isolated, mechanically disrupted and trypsinized in the presence of DNase I. Cells were then collected and plated into 24 well plates or seeded onto 13 mm round glasses, in both case pre-coated with poly-L-lysine (50 µg/mL) at a cell density of 4 × 10^5^ cells per well. Cultures were maintained at 37 °C, 5% CO_2_, 95% humidity in BME (Basal Medium Eagle, Life Technologies) supplemented with 10% fetal bovine serum, 25 mM KCl, 2 mM glutamine, and 50 µg/mL gentamicin (hereafter indicated as complete culture medium). To arrest growth of non-neuronal cells, cytosine arabinoside (10 µM) was added to the complete culture medium 18–24 h after plating.

CGNs at 6–8 days in vitro (DIV) were incubated with increasing concentrations of the indicated drugs in complete culture medium for 30 min at 37 °C; for immunofluorescence experiment, the highest concentration of inhibitor was used. Then, TeNT (2 nM) was added to the medium for 10 min, cells were washed, and culture medium with the same concentration of inhibitor was restored and incubation prolonged for 4 h at 37 °C. Finally, cells were lysed for immunoblotting or processed for immunofluorescence analysis. The specific proteolytic activity against VAMP-2 was evaluated by following the cleavage of the substrate using an antibody able to recognize only the intact form of the protein. In the immunoblotting analysis, the quantification was performed normalizing the signal on STX-1A and SNAP-25 content.

### 4.3. Western Blot

Cells were directly lysed with Laemmli sample buffer containing complete Mini EDTA-free protease inhibitors (Roche, Basel; Switzerland). Cell lysates were loaded onto NuPage 4–12% Bis-Tris gels (Life technologies, Carlsbad, CA, USA) and separated by electrophoresis in MOPS buffer (Life technologies). Proteins were transferred onto Protran nitrocellulose membranes (Whatman from Sigma Aldrich) and saturated for 1 h in PBS-T (PBS, 0.1% Tween 20) supplemented with 5% non-fatty milk. Incubation with primary antibodies (anti-SNARE Abs) was performed overnight at 4 °C. The membranes were washed three times with PBS-T and incubated with HRP-conjugated secondary antibodies for 1 h at room temperature. Membranes were washed three times with PBS and proteins revealed with chemiluminescence using Uvitec Cambridge system. VAMP-2 content was determined as a ratio with STX-1A or SNAP-25 staining, considering the value of non-treated cells as 100%.

### 4.4. Immunofluorescence Analysis

After treatment, neurons were washed with PBS and fixed for 10 min with 4% (*w/v*) paraformaldehyde in PBS. Then, cells were quenched (50 mM NH_4_Cl in PBS) for 20 min, permeabilized (5% CH_3_COOH in EtOH) for 20 min at −20 °C and incubated with the indicated primary antibodies for 2 h. Cells were washed three times with PBS, incubated with Alexa Fluorophores 488- or 555-conjugated Ab for 1 h. Coverslips were mounted using Fluorescent Mounting Medium (Dako; Santa Clara, California; USA). Cells were then analyzed by epifluorescence (Leica CTR6000; Wetzlar; Germany) microscopy and images were collected with the same lamp intensity and exposure time. TeNT cleavage was evaluated by monitoring the disappearance of VAMP-2 full-length staining. As the internal control (not shown), anti-β3-tubulin was used.

### 4.5. Mouse Bioassay

Swiss-Webster adult male CD1 mice (20–24 g) were housed under controlled light/dark conditions, and food and water were provided ad libitum. All experiments were performed in accordance with the European Community Council Directive n° 2010/63/UE and approved by the Italian Ministry of Health. Mice were preconditioned with i.p. injection of Ebselen (7.5 mg/Kg) or EGA (20 mg/Kg) or vehicle (DMSO) every 12 h for 60 h. 30 min after the last drug injection, mice were i.p. treated with TeNT (2 pg/g) corresponding to 2 × LD_50_. Toxin solution was prepared as 2 pg of toxin per µL of physiologic solution (NaCl 0.9%; gelatin 0.2%) and mice were i.p. injected with different volumes according to their body weight. After toxin injection, mice were monitored every 6 h for 15 days, when the experiment was considered concluded. A human endpoint was set when treated mice showed symptoms of tetanus, including hunched back, paralysis of rear limbs, or disappearance of the righting reflex, and these animals were euthanized with a cervical dislocation and scored positive for tetanus.

### 4.6. Statistical Analysis

For all in vitro experiments, data are shown as mean values and bars indicate the standard deviation (SD). Significance of in vivo experiments was calculated by Gehan–Breslow–Wilcoxon test. * *p* < 0.05, ** *p* < 0.01, *** *p* < 0.001, and **** *p* < 0.0001. Only values below 0.05 were considered significant.

## Data Availability

Data is contained within the article.
